# Patient satisfaction with primary healthcare services: are there any links with patients’ symptoms of anxiety and depression?

**DOI:** 10.1186/s12875-018-0780-z

**Published:** 2018-06-19

**Authors:** Rima Kavalnienė, Aušra Deksnyte, Vytautas Kasiulevičius, Virginijus Šapoka, Ramūnas Aranauskas, Lukas Aranauskas

**Affiliations:** 1 Institute of Clinical Medicine of the Faculty of medicine of Vilnius University, Clinic of Internal Diseases, Family Medicine and Oncology, Santariskiu g. 2, Vilnius, Lithuania; 2 Nordland Hospital, Parkveien, 95 Bodø, Norway; 3 Institute of Clinical Medicine of the Faculty of medicine of Vilnius University, Clinic of Psychiatry, Vasaros g.5, Vilnius, Lithuania; 4 Faculty of medicine of Vilnius University, Čiurlionio g, 21 Vilnius, Lithuania

**Keywords:** Patient satisfaction, Primary healthcare, Depression symptoms, Anxiety symptoms

## Abstract

**Background:**

The aim of our study was to determine the association of anxiety and depression symptoms, as well as the main socio-demographic factors, with patients’ satisfaction with primary healthcare services.

**Methods:**

The respondents were asked to fill out an anonymous questionnaire that included information on the patients’ gender, age, place of residence, education, ethnicity, the type of clinic they visited and the presence of chronic diseases. Patient satisfaction was evaluated by using a short version of the Patient Satisfaction Questionnaire. We also used the Hospital Anxiety and Depression Scale.

**Results:**

Poor evaluations of primary healthcare services were more characteristic of males, older patients, those living in district centres and villages, individuals with lower (secondary or lower) education levels, respondents of Russian ethnicity (compared to Lithuanian), patients with chronic diseases and higher anxiety and depression symptom scores. In the final regression analysis, better satisfaction with primary healthcare services was observed in respondents who were less depressed, of Polish ethnicity and who were living in a city rather than a village.

**Conclusions:**

Being more depressed or anxious, living in the district centre or countryside related to patients’ worse satisfaction with primary healthcare services. The results of nationality of patients and their satisfaction are ambiguous. The is strong correlation between the symptoms of depression and anxiety.

## Background

Social and demographic factors as well as the presence of illnesses affect the formation of patients’ attitudes, their satisfaction with healthcare services and their expectations related to these services. The associations between satisfaction with healthcare services and a broad spectrum of factors have been researched, yet studies on the links between patients’ mental disorders and their satisfaction with primary healthcare (PHC) services are relatively scarce. As many as 24% of patients who visit PHC physicians have mental disorders [1], and anxiety disorders and depression are among the most common of them [2, 3]. Patients with anxiety are more irritable and impatient and have greater difficulty focusing their attention [4–6], depressed patients have more difficulty participating in and managing most areas of their lives, and such patients are frequently characterized by less constructive thinking [1, 6, 7]. All of this may negatively affect the evaluation of PHC services. The negative effect of depression on certain aspects of satisfaction with PHC services is also seen in patients with such conditions as breast tumours [8], cardiac diseases [7], type-2 diabetes mellitus [9], patients undergoing dialysis [10], etc. In patients undergoing surgery for cataracts [11], in patients who have sustained traumas [12] and those with breast tumours [13], higher degrees of anxiety were associated with greater levels of dissatisfaction with healthcare services. Given the fact that depressive and anxiety disorders are among the most common mental disorders that PHC physicians face in their work [2, 3], and that these disorders affect patients’ everyday lives as they are linked to disability and poorer quality of life [14, 15], it is interesting and useful to know how these disorders along and their interaction with the main socio-demographic factors affect patients’ satisfaction with primary level healthcare services. In our study, we decided to look for a link between satisfaction with the symptoms of anxiety and depression, but not with the diagnosis of these diseases, because we believe that it will more accurately and more sensitively show the relationship between the current emotional state of the patient and the assessment of PHC services. The authors hypothesize that the presence of anxiety and depressive symptoms negatively affects the assessment of PHC services and that the expectations of patients with these symptoms are not fully fulfilled.

The aim of our study was to determine the associations of patients’ anxiety and depression symptoms, as well as the main socio-demographic factors, with patients’ satisfaction with PHC services. This study will attempt to assess whether the expectations of depressed and anxious patients are being implemented. It is expected to reveal the most problematic points for the range of services provided to such patients, whose correction would allow for the optimization and improvement of the quality of services provided for them.

## Methods

This study was carried out in Vilnius area in Lithuania, during 2016–2017. This region has a population of around 640,000 served by 63 PHC centres. As we wanted to get sufficient groups of patients with depression and anxiety symptoms (min 100 patients with each symptoms group), a sample size of 800 was appropriate. The survey of patients was carried out at 24 PHC institutions and the total number of questioned patients was 889. The questionnaire was given to patients who for various reasons visited their PHC centres and agreed to participate in the study. The main inclusion criteria were to be aged over 18 and have the ability to properly understand and fill out a questionnaire. The respondents were asked to fill out an anonymous questionnaire that included information on the patients’ gender, age, place of residence, education, ethnicity, the type of clinic they visited (state-owned or private) and the presence of chronic diseases (except for depression and anxiety, if any). Patients were asked to point their chronic diseases from the offered to them list of the diseases. This list was formed with reference to list of 16 diseases of comorbidity components of Charlson Comorbidity Index. Patient was evaluated as having chronic diseases when at least one disease was marked. The type of the clinic (state-owned or private) depended only on whether the owner of the premises was a state-financed or private individual, because healthcare services at clinics of both types are financed by state funds.

Patient satisfaction was evaluated by using an adapted short version of the Patient Satisfaction Questionnaire (PSQ-18, Marshall and Hays). The questionnaire consists of 18 closed-type questions and is used for the evaluation of patients’ satisfaction with medical services in six main domains: General Satisfaction, Technical Quality, Interpersonal Manner, Communication, Financial Aspects, Time Spent with the Doctor, and Accessibility and Convenience. We also evaluated the total sum score of all subscales. The subjects had five options when answering each question: Strongly Agree, Agree, Uncertain, Disagree and Strongly Disagree. Respondents received from 1 to 5 points for each answer, where 5 meant the highest satisfaction. According to the PSQ-18 scoring system, the sum score of all subscales may range from 18 to 90 points, where 18 points is the poorest possible evaluation and 90 points the best. To evaluate the internal consistency of the questionnaire, Cronbach’s alpha coefficient (0.96) was calculated and was found to be very good.

We also used the Hospital Anxiety and Depression Scale (HAD, Zigmond and Snaith). The scale is translated into Lithuanian and has been widely used since 1991. The HAD scale consists of 7 questions aimed at evaluating symptoms of depression (the HAD-D subscale) and 7 questions for an evaluation of symptoms of anxiety (the HAD-A subscale). The sum score in each subscale may range from 0 to 21 points. The scores indicate the severity of the symptoms of depression or anxiety: from 0 to 7 points, normal anxiety or mood; from 8 to 10 points, mild symptoms of depression or anxiety; from 11 to 14 points, moderate; and from 15 to 21 points, severe symptoms of depression or anxiety. The internal reliability and consistency of each HAD subscale was evaluated by calculating Cronbach’s alpha, which was 0.87 and was evaluated as good.

Statistical data analysis was conducted using the Statistical Package for Social Sciences (SPSS), version 24.0. A database was created for the processing of questionnaire data. To set coding and input errors, we used the procedure ‘frequencies’, where we set the minimum and maximum values for each of the answers and if they exceeded the ones contained in the questionnaire, we corrected them according to the source material. We also used ‘frequencies’ for the response frequency calculations.

For the statistical distribution of the frequency of responses between the discrete (nominal and ordinal) data the non-parametric Pearson’s chi square (χ^2^) criterion is chosen with 95% probability, i.e., the difference is considered statistically reliable at *p* < 0.05. For determining the strength and direction of statistical correlations between data the non-parametric Spearman correlation coefficient is chosen, when p < 0.05. Using the Spearman coefficient does not matter if the variable values are symmetric. In the results with means, the standard deviation from the mean (SD) was also indicated.

The One Way ANOVA test was used to compare the means of total PSQ-18 scores, PSQ-18 subscales’ scores as well as HAD subscales’ scores according to patients’ socio-demographic factors; Post-Hoc tests were used to compare three or more groups. The effect of all demographic and social factors and anxiety and depression symptoms on patient satisfaction and its strength was assessed using an ordinal regression analysis. As a dependent ordinal variable, we used the evaluation of patients’ satisfaction, which we obtained by splitting the overall PSQ-18 score into three ranks: good (PSQ-18 score from 90 to 66), medium (from 65 to 42) or poor (from 41 to 18) evaluation. In the education, nationality and place of residence groups, we used secondary and lower education, Lithuanian nationality and urban life as comparable variables, because we were expecting the biggest differences in satisfaction with these groups.

The study proposal was approved by the Regional Biomedical Research Ethics Committee in Vilnius, Lithuania.

## Results

In total, 889 patients filled out the questionnaire. Two questionnaires were excluded from the analysis because of filling errors or incomplete filling, and thus 887 questionnaires were included in the data analysis. The respondents’ distribution according to their socio-demographic and other characteristics is presented in Table 1.

**Table 1:** Respondents’ distribution according to their socio-demographic and other characteristics

Respondents’ characteristics	Number of respondents	Percent
Gender		
Male	356	40.1%
Female	531	59.9%
Age group		
18–40 years	298	33.6%
41–60 years	388	43.7%
61–87 years	201	22.7%
Living area		
City	715	80.6%
District centre	78	8.8%
Village	94	10.6%
Education level		
Secondary or lower	280	31.6%
Specialized secondary	151	17.0%
Higher	429	48.4%
Other	27	3%
Ethnicity		
Lithuanian	520	58.6%
Russian	173	19.5%
Polish	175	19.7%
Other	19	2.1%
Type of PHC clinic		
State-owned	685	77.2%
Private	202	22.8%
Chronic diseases		
Yes	511	57.6%
No	376	42.4%
Total	887	100.0%

### Associations according to patient satisfaction questionnaire (PSQ-18)

The total PSQ-18 scores ranged from 21 to 90 points, the mean score being 59.9 ± 14.6 points. The majority, i.e. 44% of the patients, evaluated the PHC services as good (90 to 66 points), 38% of the respondents gave average evaluations (65 to 42 points) and the lowest percentage of respondents (18%) evaluated PHC services poorly (41 to 0 points). Poor evaluations of PHC services were more characteristic of males, older patients, those living in district centres and villages, individuals with lower (secondary or lower) education levels, respondents of Russian ethnicity (compared to Lithuanian) and patients with chronic diseases (ρ < 0.05). Patients of state-owned clinics more frequently presented average evaluations, patients of private clinics good evaluations, while the frequency of poor evaluations did not differ between these two groups.

The most positive evaluations were observed in the Interpersonal Manner and Communication subscales (mean scores 3.59 and 3.55, respectively). The poorest evaluations were observed in the statements of the Accessibility and Convenience and Time Spent with the Doctor subscales (mean scores 2.97 and 3.20, respectively).

### Associations according to depression and anxiety symptoms

The evaluation of the scores of the HAD scale showed that 290 (32.7%) patients had symptoms of anxiety of any level, and 193 (21.8%) had symptoms of depression of any level. The frequency of the levels of the severity of symptoms of depression and anxiety among the subjects is presented in Table 2. The results obtained showed that 88% of all patients who had symptoms of depression of any level also had symptoms of anxiety, and 58.6% of all patients with anxiety had symptoms of depression as well. Both subscales of anxiety and depression strongly correlated with each other (Spearman’s correlation coefficient 0.742; ρ < 0.001).

**Table 2:** Frequency of respondents’ levels of severity of symptoms of depression and anxiety

Level of severity of symptoms	Anxiety scale		Depression scale	
	Number of respondents	%	Number of respondents	%
Normal mood	597	67.3%	694	78.2%
Mild symptoms	146	16.5%	106	12%
Moderate symptoms	127	14.3%	63	7.1%
Severe symptoms	17	1.9%	24	2.7%
Total	887	100%	887	100%

Higher scores in the depression subscale were associated with male gender, older age, living in a district centre or village, poorer education, the presence of chronic diseases and being of Russian or Polish rather than Lithuanian ethnicity (ρ < 0.015). Meanwhile, higher scores in the anxiety subscale were associated only with older age (Spearman’s correlation coefficient 0.228; ρ < 0.001) and the presence of chronic diseases (ρ < 0.001).

Poorer scores in both HAD subscales correlated with a poorer total PSQ-18 score and poorer scores in all subscales (ρ < 0.001) (Table 3).

**Table 3:** Spearman’s correlation coefficients between Hospital Anxiety and Depression and Patient Satisfaction Questionnaire (Short Form) scores

			HAD-Anxiety score	HAD-Depression score
Spearman’s rho	General Satisfaction	Correlation Coefficient	**−.421**	**−.470**
		Sig. (2-tailed)	.000	.000
	Technical Quality	Correlation Coefficient	**−.368**	**−.430**
		Sig. (2-tailed)	.000	.000
	Interpersonal Manner	Correlation Coefficient	**−.356**	**−.405**
		Sig. (2-tailed)	.000	.000
	Communication	Correlation Coefficient	**−.390**	**−.453**
		Sig. (2-tailed)	.000	.000
	Financial Aspects	Correlation Coefficient	**−.432**	**−.490**
		Sig. (2-tailed)	.000	.000
	Time Spent with Doctor	Correlation Coefficient	**−.390**	**−.467**
		Sig. (2-tailed)	.000	.000
	Accessibility and Convenience	Correlation Coefficient	**−.446**	**−.521**
		Sig. (2-tailed)	.000	.000
	Total PSQ-18 score	Correlation Coefficient	**−.462**	**−.536**
		Sig. (2-tailed)	.000	.000
		N	887	887

### Regression analysis

In the beginning, we carried out ordinal regression analysis only with the socio-demographic factors and patient satisfaction with PHC services (Nagelkerke = 0.100) (Table 4). We saw a link between the assessment of better satisfaction with the younger age of respondents, special secondary education, Polish nationality and the place of residence in the city rather than in the district centre or village.

**Table 4:** Ordinal regression analysis of Patient Satisfaction Questionnaire (Short Form) evaluation and respondents’ socio-demographic factors as well as type of clinic (Nagelkerke = 0.100)

Respondents’ characteristics	Estimate	Std.Error	Wald	Sig.	95% Confidence interval	
					Lower Bound	Upper Bound
Age	−.024	.005	27.334	**.000**	−.033	−.015
Gender:						
Male	0^a^	–	–	–	–	–
Female	−.090	.137	.436	.509	−.358	.177
Education level:						
Secondary or lower	0^a^	–	–	–	–	–
Specialized secondary	−.531	.200	7.034	**.008**	−.923	−.138
Higher	.114	.163	.488	.485	−.206	.434
Ethnicity:						
Lithuanian	0^a^	–	–	–	–	–
Russian	.071	.186	.146	.703	−.294	.436
Polish	−.460	.190	5.881	**.015**	−.832	−.088
Living area:						
City	0^a^	–	–	–	–	–
District centre	.769	.239	10.306	**.001**	.299	1.238
Village	1.326	.242	30.104	**.000**	.853	1.800
Type of the clinic:						
State owner	0^a^	–	–	–	–	–
Private owner	−.211	.158	1.785	.182	−.520	.098

^a^Parameter set to zero because it is redundant

Since there was a strong inter-correlation between both HAD subscales (symptoms of anxiety and depression), we carried out separate regression analyses with each subscale and subsequently joined both HAD subscales and evaluated the joint result. We also added the presence of chronic diseases if any. In regression analysis with the HAD depression subscale and socio-demographic factors, a better total PSQ-18 score correlated with lower depression scores, Polish ethnicity and living in a city rather than a village (Nagelkerke = 0.385) (Table 5). In regression analysis with the HAD anxiety subscale and socio-demographic factors, a better total PSQ-18 score correlated with Polish ethnicity, living in a city rather than a village and in a district centre, a younger age and better education (Nagelkerke = 0.276) (Table 6). In the final variant, after the inclusion of both HAD subscales into the regression analysis, better satisfaction with PHC services was observed in respondents who were less depressed, of Polish ethnicity and who were living in a city rather than a village (Nagelkerke = 0.361) (Table 7).

**Table 5:** Ordinal regression analysis of Patient Satisfaction Questionnaire (Short Form) evaluation and respondents’ characteristics (with HAD-Depression subscale) (Nagelkerke = 0.385)

Respondents’ characteristics	Estimate	Std.Error	Wald	Sig.	95% Confidence interval	
					Lower Bound	Upper Bound
HAD-Depression	−.329	.024	185.725	**.000**	−.376	−.281
Age	.005	.006	.698	.404	−.007	.017
Chronic diseases:						
No	0^a^	–	–	–	–	–
Yes	−.082	.191	.185	.667	−.455	.291
Gender:						
Male	0^a^	–	–	–	–	–
Female	−.010	.147	.004	.948	−.298	.278
Education level:						
Secondary or lower	0^a^	–	–	–	–	–
Specialized secondary	−.328	.218	2.269	.132	−.754	.099
Higher	.164	.180	.832	.362	−.188	.516
Ethnicity:						
Lithuanian	0^a^	–	–	–	–	–
Russian	−.123	.204	.364	.547	−.523	.277
Polish	−.686	.208	10.877	**.001**	−1.094	−.278
Living area:						
City	0^a^	–	–	–	–	–
District centre	.309	.265	1.360	.243	−.210	.829
Village	1.171	.259	20.431	**.000**	.663	1.678

^a^Parameter set to zero because it is redundant

**Table 6:** Ordinal regression analysis of Patient Satisfaction Questionnaire (Short Form) evaluation and respondents’ characteristics (with HAD-Anxiety subscale) (Nagelkerke = 0.276)

Respondents’ characteristics	Estimate	Std.Error	Wald	Sig.	95% Confidence interval	
					Lower Bound	Upper Bound
HAD-Anxiety	−.233	.023	106.876	**.000**	−.277	−.189
Age	−.014	.006	6.177	**.013**	−.025	−.003
Chronic diseases:						
No	0^a^	–	–	–	–	–
Yes	−.096	.187	.267	.605	−.462	.269
Gender:						
Male	0^a^	–	–	–	–	–
Female	−.138	.143	.927	.336	−.418	.142
Education level:						
Secondary or lower	0^a^	–	–	–	–	–
Specialized secondary	−.604	.210	8.308	**.004**	−1.015	−.193
Higher	.082	.174	.224	.636	−.259	.424
Ethnicity:						
Lithuanian	0^a^	–	–	–	–	–
Russian	−.157	.194	.653	.419	−.224	.537
Polish	−.532	.201	6.996	**.008**	−.927	−.138
Living area:						
City	0^a^	–	–	–	–	–
District centre	.599	.254	5.576	**.018**	.102	1.096
Village	1.283	.251	26.184	**.000**	.792	1.774

^a^Parameter set to zero because it is redundant

**Table 7:** Ordinal regression analysis of Patient Satisfaction Questionnaire (Short Form) evaluation and respondents’ characteristics (with both HAD-Depression and HAD-Anxiety subscales) (Nagelkerke = 0.361)

Respondents’ characteristics	Estimate	Std.Error	Wald	Sig.	95% Confidence interval	
					Lower Bound	Upper Bound
HAD-Anxiety	−.042	.029	2.208	.137	−.098	.014
HAD-Depression	−.300	.029	103.723	**.000**	−.358	−.243
Age	.006	.006	1.194	.274	−.005	.018
Chronic diseases:						
No	0^a^	–	–	–	–	–
Yes	−.244	.181	1.808	.179	−.599	.111
Gender:						
Male	0^a^	–	–	–	–	–
Female	−.004	.144	.001	.977	−.286	.278
Ethnicity:						
Lithuanian	0^a^	–	–	–	–	–
Polish	−.505	.186	7.372	**.000**	−.869	−.140
Living area:						
City	0^a^	–	–	–	–	–
District centre	.272	.258	1.115	.291	−.233	.777
Village	.768	.235	10.622	**.001**	.305	1.229

^a^Parameter set to zero because it is redundant

## Discussion

Our study shows that depressed and anxious patients had a negative opinion about healthcare services, and that was visible in all PSQ-18 subscale estimates.

The PSQ-18 and HAD subscales had the strongest coefficient correlation in the subscales General Satisfaction, Financial Aspects, Accessibility and Convenience, and Time Spent with Doctor. Similar results were obtained by Bair et al. [16]. They investigated the link between the diagnosed depression and patients’ satisfaction with PHC services. The authors believe that depressed and anxious patients do not receive suitable psychological aid due to a relatively short consultation time. Moreover, they think that depressed patients receive PHC services more often, thus, they are more sensitive to the long waiting time. In order to examine the satisfaction of psychiatric outpatients with the health care services, Holikatti et al. also employed PSQ-18 questionnaire [17]. Their findings show that outpatients with Major Depressive Disorder and Anxiety Disorder were the most disappointed with the Financial Aspects and Communications.

We observed a strong connection between the symptoms of depression and anxiety. This relation is perceived by many scholars [6, 18–20]. In our research, the symptoms of depression had a stronger influence on the outpatients‘ satisfaction than the symptoms of anxiety. Contrarily, Holikatti et al. found that the anxiety has a stronger influence on the satisfaction than depression and leads to worse satisfaction estimates [17].

We noticed that the nationality of patients affects the satisfaction of the services; however, this influence is still ambiguous. The majority of the subscales were evaluated the best by the Lithuanians. The satisfaction with the technical quality only received the higher evaluation from the Polish; nonetheless, this finding lacks the statistical reliability. The satisfaction of the Russians was very poor in all subscales. Typically, the ethnic minorities assessed the health care services worse than the national population. This could be caused by the lower reliance on the medical personnel [21], the self-isolation of the ethnic groups, and the longstanding political tendency to view themselves as different from the local majority [22].

We found that the residency in the city causes a better satisfaction with services in all PSQ-18 subscales. The same results were also noticed in the pilot study [23]. As the inhabitants of villages are less educated and may have difficulties reaching the physician, the residency in the rural areas could be a reason for the poorer satisfaction with PHC services.

Generally, during the evaluation of PSQ-18 estimates, we noticed the remarkably low satisfaction estimates of Accessibility and Convenience, and Time Spent with Doctor. Such finding is confirmed by other studies, which found a correlation between the poor accessibility of PHC services, shorter consultation time and poorer satisfaction with the received PHC services [24, 25]. Michael et al. state that the satisfaction with PHC services improves if the waiting time before a consultation is shortened [26]. The authors suggest certain work methodologies, which would permit the reduction of the waiting time before a consultation. Anderson et al. found that the shorter consultation time was the strongest prognostic index causing poorer satisfaction [27]. These authors suggest that if the consultation time was extended by at least 5 min, the patients would be more satisfied not only with the consultation, but also with the waiting time before a consultation. The availability of services in our research was slightly higher only in the private PHC clinics. Andersen et al. notice that the private clinics optimize non-clinical factors such as waiting time much more than the public ones [28]. Private clinics do not achieve noticeably better clinical results, thus the total patient satisfaction with private clinics is not always higher [29].

Recommendations: In order to improve the patient satisfaction with PHC services, the authors suggest the screening for the anxiety and depression symptoms among their patients; if found, those symptoms should be reduced. When the anxiety and depression symptoms are reduced, the satisfaction with the health care services can be expected to increase.

The increased satisfaction with the provided PHC services is important as the patients satisfied with the consultations are more likely to adhere to their treatment plan, take a better care of their health and lead a healthy lifestyle. This determines better outcomes of their treatment [24]. In order to reach such goals, it is important to give information to patients with the symptoms of anxiety and depression about medical and social assistance, and psychological support opportunities. It is also important to regularly develop the emotional support and cooperation skills for students of medicine and employees of family centers. These skills should be actively used during the problem solving of the depressed and anxious patients.

The advantages of this study are a reasonably large survey sample, and the usage of PSQ-18 and HAD scales together in the primary health care level research. The fact that research only included residents of one region of a country presents the limitation of our study; the situation further away from the capital city may be different. Also, patients who did not understand the national language could not complete the questionnaire; the opinion of ethnic minority patients, who are only native speakers, could be different also.

## Conclusions

The worse satisfaction with PHC services is associated with the higher degree of outpatients‘ symptoms of depression and anxiety, and their residency in the district centre or countryside; the found association between the nationality of patients and their satisfaction with PHC services was ambiguous. The symptoms of depression and anxiety of outpatients strongly correlate between each other. The satisfaction with the PHC services was more influenced by social and cultural (place of residence or nationality) than by demographic (age and gender) factors. The authors recommend the active patient screening for symptoms of depression and anxiety, and their reduction, in order to improve the satisfaction with PHC services.

## Abbreviations

HAD: Hospital Anxiety and Depression Scale
HAD–A: Hospital Anxiety and Depression Scale’s Anxiety Subscale
HAD-D: Hospital Anxiety and Depression Scale’s Depression Subscale
PHC: Primary Healthcare
PSQ-18: Patient Satisfaction Questionnaire, Short Version
